# Engineering topological chiral transport in a flat-band lattice of ultracold atoms

**DOI:** 10.1038/s41377-025-02025-3

**Published:** 2025-09-17

**Authors:** Hang Li, Qian Liang, Zhaoli Dong, Hongru Wang, Wei Yi, Jian-Song Pan, Bo Yan

**Affiliations:** 1 Zhejiang Key Laboratory of Micro-nano Quantum Chips and Quantum Control, School of Physics, and State Key Laboratory for Extreme Photonics and Instrumentation, Zhejiang University, Hangzhou, China; 2 Laboratory of Quantum Information, University of Science and Technology of China, Hefei, China; 3 Anhui Province Key Laboratory of Quantum Network, University of Science and Technology of China, Hefei, China; 4 CAS Center For Excellence in Quantum Information and Quantum Physics, Hefei, China; 5 College of Physics and Key Laboratory of High Energy Density Physics and Technology of Ministry of Education, Sichuan University, Chengdu, China

**Keywords:** Atom optics, Atom optics

## Abstract

The manipulation of particle transport in synthetic quantum matter is an active research frontier for its theoretical importance and potential applications. Here we experimentally demonstrate an engineered topological transport in a synthetic flat-band lattice of ultracold ^87^Rb atoms. We implement a quasi-one-dimensional rhombic chain with staggered flux in the momentum space of the atomic condensate and observe biased local oscillations that originate from the flat-band localization under the staggered synthetic flux. Based on these features, we design and experimentally confirm a state-dependent chiral transport under the periodic modulation of the synthetic flux. We show that the phenomenon is associated with the topology of the Floquet Bloch bands of a coarse-grained effective Hamiltonian. Our work opens the new avenue for exploring flat-band-assistant topological transport with ultracold atoms, and offers a new strategy for designing efficient quantum device with topological robustness.

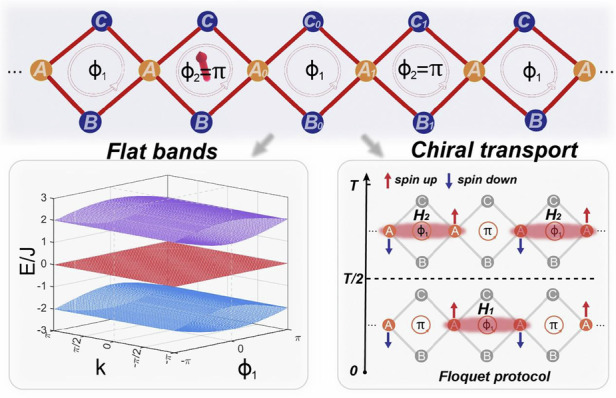

## Abstract (web summary)

We demonstrate topological chiral transport in an inherently flat-band-localized system, enabled by the elegant engineering of a helical Floquet channel via flexible control of synthetic gauge fields.

## Introduction

Quantum control is an essential ingredient of modern quantum science and technology. The quest of manipulating localization and transport dynamics, in particular, has significantly enriched our understanding of quantum matter^1–3^, with ample implications for quantum device design^4–10^. In the region of cold atoms, the development of optical lattices greatly facilitates the exploration of topological transport^11–13^ in real-space lattice potentials. Compared to conventional optical lattices, synthetic momentum lattices feature flexible lattice design, reduced experimental complexity, and highly malleable parameters such as the site-resolved hopping patterns, quasiperiodicity and disorder, as well as variant forms of synthetic gauge fields. The momentum lattice is therefore an important platform for simulating and exploring transport dynamics in a wealth of physical contexts^14,15^. Over the past decade, momentum-lattice engineering has helped to reveal rich quantum dynamic phenomena regarding topological matter^16,17^, non-Hermitian physics^18,19^, correlated dynamics in frustrated geometry^20^, as well as localization and mobility edges^21–23^. A prime achievement here is the experimental demonstration of the inverse Anderson localization^24–28^, where disorder counterintuitively enhances delocalization in flat-band lattices via Aharonov-Bohm (AB) caging mechanisms^29–32^. Recently implemented in photonics^33,34^ and cold atoms in twisted optical lattices^35^, systems with flat bands exhibit striking localization and transport properties due to their dispersionless energy spectra. Despite these advances, a critical question remains–whether the transport properties of flat-band lattices can be dynamically tuned or switched between localized and delocalized regimes–a challenge central to quantum manipulation and simulation.

In this work, we experimentally demonstrate chiral transport in a synthetic rhombic chain in the momentum space of ultracold ^87^Rb atoms. Adopting the state-of-the-art momentum-lattice engineering technique, we are able to switch and tune the synthetic flux threaded through each plaquette on demand and probe the dynamics of the condensate along the synthetic lattice. We observe local breathing modes and biased oscillations in a binary staggered-flux configuration, both characteristic of the flat-band localization under the staggered flux. We then engineer a state-dependent chiral transport by introducing Floquet modulations to the flux. While the micromotion of the Floquet dynamics involves the occupation of multiple sublattice sites and is complicated in general, the observed chiral transport is consistent with the quantized winding of the Floquet Bloch bands of a coarse-grained effective Hamiltonian. While the topological robustness and fast time scale of the quantized transport can be useful for quantum device design, our setup further paves the way for the exploration of the rich dynamics under the impact of the flat-band localization, disorder, Floquet driving, and long-range interactions typical of the momentum lattice. To the best of our knowledge, the observed topological transport in a system with flat-band localization is the first of its kind and absent in all existing studies.

## Results

### Implementing the staggered-flux lattice

In our experiment, the flat bands are induced by implementing synthetic fluxes in a rhombic chain. The manipulation of synthetic flux in ultracold atomic systems is a powerful tool for manipulating band structures and dynamics, as well as for simulating and studying topological phases. For instance, synthetic flux plays key roles in generating flat bands in the AB cage^26^ and the Creutz lattice^36^, where particles exhibit highly localized states due to interference. Synthetic flux is also crucial for the simulation of the Hofstadter model^37^, which features fractal spectra and topological bands. The interplay of different forms of synthetic flux, lattice geometry and interaction can also give rise to dynamic phases and transitions^20,38–40^. In open systems, the combination of synthetic flux and local loss leads to directional dynamics signaling important non-Hermitian effects^19^.

**Fig. 1:**
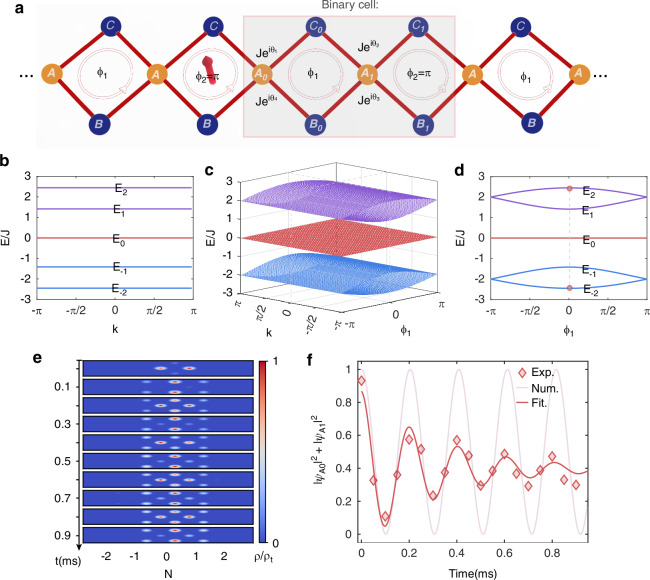
Schematic of the rhombic flux-staggered chain and the breathing mode. **a** Schematics of a rhombic flux-staggered chain with flexibly programmable artificial gauge fields. The subfigure shows the binary flux-staggered ladder configuration with a static synthetic flux setting. The complex hopping coefficients between the nearest-neighbor sites are denoted as $J{e}^{i{\theta }_{m}}(m=1,2,3,4)$. **b**–**d** The energy dispersion *E*(*k*) of the binary-flux lattice chain with *ϕ*_2_ = *π*. All the bands display complete flat-band features no matter what *ϕ*_1_ is. **b** When *ϕ*_1_ ≠ ± *π*, the binary-flux lattice has six flat bands, labeled as *E*_±2_, *E*_±1_, *E*_0_ respectively. The central band with *E*_0_ is two-fold degenerate. Here we choose *ϕ*_1_ = 0, under which the splitting of the upper and lower bands is the largest. **d** The flat-band splitting with the variation of *ϕ*_1_. **e** The evolution dynamics for an initial state $\left\vert {\psi }_{{{\rm{ini}}}}\right\rangle =\frac{1}{\sqrt{2}}({\hat{a}}_{n}^{{\dagger} }+{\hat{a}}_{n+1}^{{\dagger} })\left\vert 0\right\rangle$ with the binary-flux parameter {*ϕ*_1_ = 0, *ϕ*_2_ = *π*}. The color map represents the measured atom density *ρ*/*ρ*_*t*_, normalized by the total density *ρ*_*t*_ at each discrete time slice. **f** Measuring a damped oscillation of the total population of sites *A*_0_ and *A*_1_ around 0.9 ms. The red solid lines represent the fitting of experimental data, and the pale red line is the numerical results calculated using *H*_eff_ in Eq. (1)

Here we consider a binary-flux-ladder (BFL) configuration with recurring synthetic fluxes *ϕ*_1_ and *ϕ*_2_, as illustrated in Fig. 1a. Each unit cell contains 6 sublattice sites, respectively labeled {*A*_2*j*_, *B*_2*j*_, *C*_2*j*_, *A*_2*j*+1_, *B*_2*j*+1_, *C*_2*j*+1_} in the *j*-th unit cell. Under the tight-binding approximation^41,42^, the system is described by the effective Hamiltonian

1 $$\begin{array}{rcl}{H}_{{{\rm{eff}}}}&=&\mathop{\sum}\limits_{j}\left[-J\left({e}^{i{\theta }_{2j-1,3}}{\hat{a}}_{2j}^{{\dagger} }{\hat{b}}_{2j-1}+{e}^{-i{\theta }_{2j,4}}{\hat{a}}_{2j}^{{\dagger} }{\hat{b}}_{2j}\right.\right.\\ &&+{e}^{-i{\theta }_{2j-1,2}}{\hat{a}}_{2j}^{{\dagger} }{\hat{c}}_{2j-1}+{e}^{i{\theta }_{2j,1}}{\hat{a}}_{2j}^{{\dagger} }{\hat{c}}_{2j}+{e}^{i{\theta }_{2j,3}}{\hat{a}}_{2j+1}^{{\dagger} }{\hat{b}}_{2j}\\ &&+{e}^{-i{\theta }_{2j+1,4}}{\hat{a}}_{2j+1}^{{\dagger} }{\hat{b}}_{2j+1}+{e}^{-i{\theta }_{2j,2}}{\hat{a}}_{2j+1}^{{\dagger} }{\hat{c}}_{2j}+\\ &&\left.\left.{e}^{i{\theta }_{2j+1,1}}{\hat{a}}_{2j+1}^{{\dagger} }{\hat{c}}_{2j+1}+{{\rm{H}}}.{{\rm{c}}}.\right)\right]\end{array}$$

where *J* is the nearest-neighbor hopping rate, ${\hat{a}}_{2j/2j+1}^{{\dagger} }$, ${\hat{b}}_{2j/2j+1}^{{\dagger} }$ and ${\hat{c}}_{2j/2j+1}^{{\dagger} }$ (${\hat{a}}_{2j/2j+1}$, ${\hat{b}}_{2j/2j+1}$ and ${\hat{c}}_{2j/2j+1}$) are the creation (annihilation) operators for atoms on the sublattice sites of the *j*-th unit cell, respectively. Here {*A*_*n*_, *B*_*n*_, *C*_*n*_, *A*_*n*+1_} encircle the *n*-th plaquette, consistent with Fig. 1a. Since the synthetic flux of the *n*-th plaquette *ϕ*_*n*_ is given by ${\phi }_{n}(t)=\mathop{\sum }_{m = 1}^{4}{\theta }_{n,m}(t)$, we henceforth adopt the gauge convention: *θ*_*n*,2_ = *ϕ*(*t*), and *θ*_*n*,*m*_ = 0 otherwise.

The band dispersions of Hamiltonian (1) are *E*_0_ = 0, ${E}_{\pm 1}=\pm J\sqrt{4-\sqrt{2F({\phi }_{1},{\phi }_{2})}}$, and ${E}_{\pm 2}=\pm J\sqrt{4+\sqrt{2F({\phi }_{1},{\phi }_{2})}}$. Here the flux-dependent factor $F({\phi }_{1},{\phi }_{2})=2+\cos ({\phi }_{1})+\cos ({\phi }_{2})+4\cos (2k)\cos ({\phi }_{1}/2)\cos ({\phi }_{2}/2)$, *k* (− *π*≤*k* < *π*) is the Bloch wave number. For convenience, we have set the lattice constant to unity. Notably, when *ϕ*_2_ = *π*, $F({\phi }_{1},{\phi }_{2})=2{\cos }^{2}({\phi }_{1}/2)$ for arbitrary *ϕ*_1_. All the bands are then flat regardless of *ϕ*_1_ [see Fig. 1b–d]. When *ϕ*_1_ ≠ ± *π*, the binary-flux lattice has six flat bands with the central band being two-fold degenerate. As shown in Fig. 1b, we label these flat-bands as *E*_±2_, *E*_±1_, *E*_0_ respectively. Through changing the values of *ϕ*_1_, we can adjust the splitting between *E*_±2_ and *E*_±1_ as shown in Fig. 1d. This gives rise to intricate localized states and opens up room for engineering the transport dynamics, as we show below.

Experimentally, we implement the rhombic chain in Fig. 1a along a Raman-coupled momentum lattice in a ^87^Rb Bose-Einstein condensate (BEC), which contains ~ 2 × 10^5^ atoms^43–45^. As illustrated in Fig. 1a, sublattice sites {*A*_*n*_, *B*_*n*_, *C*_*n*_} are encoded in the momentum states of atomic hyperfine levels (see Methods): *A*_*n*_ and *B*_*n*_ correspond to different momentum states of $\left\vert F=1,{m}_{F}=0\right\rangle$, and *C*_*n*_ is encoded in those of $\left\vert F=2,{m}_{F}=0\right\rangle$, respectively. Hopping between adjacent momentum-lattice sites is implemented by two-photon Raman or Bragg processes^26,46^, with a fixed hopping amplitude *J*/*h* = 0.95(5) kHz. The flux *ϕ*_*n*_ is meticulously controlled by locking the relative phases of the coupling lasers. The spatial (and temporal later) inhomogeneity of the flux pattern requires a much-improved stability in phase locking, compared to previous studies with a homogeneous flux^26^. More details about implementing the Raman lattice are illustrated in the “Methods” section and Supplementary materials^46^.

### Breathing mode and biased oscillation

An outstanding dynamic signature of the flat-band configuration is the localized breathing mode under the AB-caging mechanism^26,47–49^. The conventional AB caging occurs for *ϕ*_1_ = *ϕ*_2_ = *π* but persists under the BFL configurations with only *ϕ*_2_ = *π*. We experimentally confirm this by focusing on the case with *ϕ*_1_ = 0 and *ϕ*_2_ = *π*, which has the largest gap between the low- and high-lying bands *E*_±2_. The corresponding localized eigenstates of the two flat bands are $\left\vert {\psi }_{n,\pm 2}\right\rangle =\frac{1}{2}[({\hat{a}}_{n}^{{\dagger} }+{\hat{a}}_{n+1}^{{\dagger} })\pm \frac{\sqrt{6}}{6}(2{\hat{b}}_{n}^{{\dagger} }+2{\hat{c}}_{n}^{{\dagger} }-{\hat{b}}_{n-1}^{{\dagger} }+{\hat{c}}_{n-1}^{{\dagger} }+{\hat{b}}_{n+1}^{{\dagger} }+{\hat{c}}_{n+1}^{{\dagger} })]\left\vert 0\right\rangle$. As such, an initial state $\left\vert {\psi }_{{{\rm{ini}}}}\right\rangle =\frac{1}{\sqrt{2}}({\hat{a}}_{n}^{{\dagger} }+{\hat{a}}_{n+1}^{{\dagger} })\left\vert 0\right\rangle$ can be expressed as an equal-weight superposition of $\left\vert {\psi }_{n,\pm 2}\right\rangle$ and the subsequent breathing mode should acquire a frequency proportional to the energy gap between the bands. Note that similar phenomena have been observed in photonic systems^50,51^.

We prepare the initial state $\left\vert {\psi }_{{{\rm{ini}}}}\right\rangle =\frac{1}{\sqrt{2}}({\hat{a}}_{0}^{{\dagger} }+{\hat{a}}_{1}^{{\dagger} })\left\vert 0\right\rangle$, by coherently splitting the initial BEC on site *B*_0_ onto sites *A*_0_ and *A*_1_, and detect the density dynamics as shown in Fig. 1e. While the ensuing dynamics is localized and centered around the initial site, the breathing-mode character is clearly visible in Fig. 1f. The observed breathing mode features a frequency *ω* = 2*π* × 4.9(2) kHz, consistent with the numerically calculated energy gap *ω* = 2*π* × 4.9*J* in Fig. 1d. Due to the decoherence of the Raman-coupling processes, the overall dynamics is damped, with a fitted decay lifetime of 0.40(5)ms, as illustrated in Fig. 1f. The limited lifetime of decoherence is mainly caused by the inhomogeneous interactions of trap potential and the phase noise of Raman-Bragg lasers^46^.

**Fig. 2:**
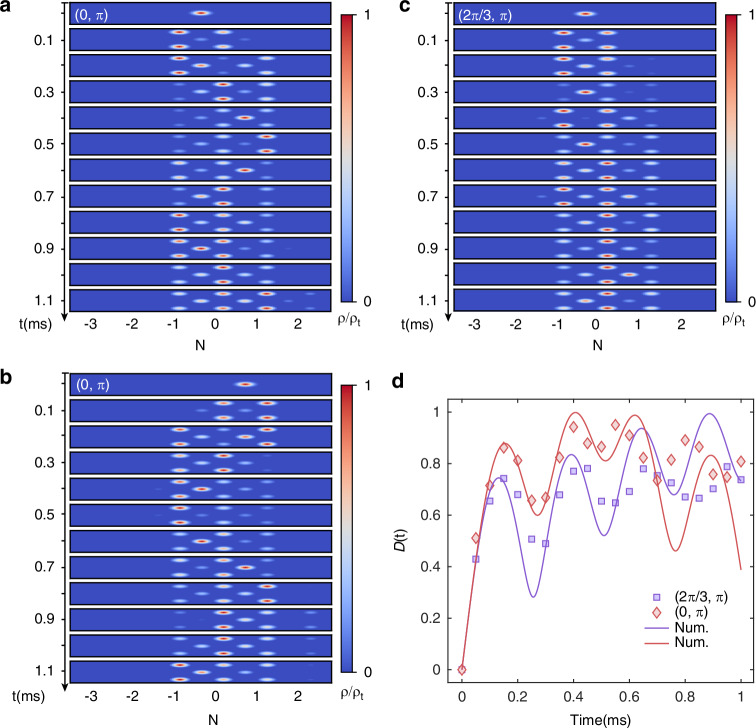
Biased oscillations in the binary flux-staggered lattice. **a** The evolution dynamics under {*ϕ*_1_ = 0, *ϕ*_2_ = *π*}, with the initial state $\left\vert {\psi }_{{{\rm{ini}}}}\right\rangle ={\hat{a}}_{0}^{{\dagger} }\left\vert 0\right\rangle$. **b** The evolution dynamics under {*ϕ*_1_ = 0, *ϕ*_2_ = *π*} with $\left\vert {\psi }_{{{\rm{ini}}}}\right\rangle ={\hat{a}}_{1}^{{\dagger} }\left\vert 0\right\rangle$. **c** The evolution dynamics under {*ϕ*_1_ = 2*π*/3, *ϕ*_2_ = *π*} with $\left\vert {\psi }_{{{\rm{ini}}}}\right\rangle ={\hat{a}}_{0}^{{\dagger} }\left\vert 0\right\rangle$. **d** The extracted ${{\mathcal{D}}}(t)$ with $\left\vert {\psi }_{{{\rm{ini}}}}\right\rangle ={\hat{a}}_{0}^{{\dagger} }\left\vert 0\right\rangle$ in different BFL parameters setting. The dots are experimental data, and solid lines are numerical simulations using *H*_eff_

For a more general choice of *ϕ*_1_ and local initial state, the dynamics of the condensate center of mass becomes oscillatory but is biased in direction depending on the initial state. For instance, Fig. 2a, b display the density dynamics under {*ϕ*_1_ = 0, *ϕ*_2_ = *π*} with different initial-site excitations. While the dynamics is largely localized within the initial unit cell under the flat-band condition (enforced by *ϕ*_2_ = *π*), atoms initialized on-site *A*_2*j*_ (*A*_2*j*+1_) move toward the right (left) at the beginning of the time evolution, and the overall oscillation is accordingly biased in direction. The biased oscillation can be interpreted as an interference effect between the upper and lower legs of the rhombic plaquette, which is fully destructive (constructive) under the *π* (0) flux. As a result, the initial condensate prepared on-site *A*_2*j*_ (*A*_2*j*+1_) tends to delocalize on the 0-flux plaquette, moving to the right (left) at short times.

Further, by varying *ϕ*_1_, we can adjust the oscillated dynamics of the local oscillation, as shown in Fig. 2c. This becomes clearer in Fig. 2d, where we plot the evolution of second moment of the position operator, defined through ${{\mathcal{D}}}(t)={\left[{\sum }_{n}{(n-{n}_{0})}^{2}(\left\langle {a}_{n}^{{\dagger} }{a}_{n}\right\rangle +\left\langle {b}_{n}^{{\dagger} }{b}_{n}\right\rangle +\left\langle {c}_{n}^{{\dagger} }{c}_{n}\right\rangle )\right]}^{\frac{1}{2}}$, where *n*_0_ is the position of initial injection state, $\left\langle {a}_{n}^{{\dagger} }{a}_{n}\right\rangle$, $\left\langle {b}_{n}^{{\dagger} }{b}_{n}\right\rangle$ and $\left\langle {c}_{n}^{{\dagger} }{c}_{n}\right\rangle$ are the expectation values of particle number operators, respectively. We further extract the two frequency components Fig. 2d, with *ω*_1_ = 2*π* × 1.4(1) kHz, *ω*_2_ = 2*π* × 2.6(1) kHz for the {*ϕ*_1_ = 0, *ϕ*_2_ = *π*} case and *ω*_1_ = 2*π* × 1.7(1) kHz, *ω*_2_ = 2*π* × 2.3(1) kHz for the {*ϕ*_1_ = 2*π*/3, , *ϕ*_2_ = *π*} case, respectively. These are consistent with the theoretical prediction of $(\left\vert {E}_{\pm 2}\right\vert \pm \left\vert {E}_{\pm 1}\right\vert )/h$^46^, explaining the *ϕ*_1_ dependence of the biased oscillation.

### Topological chiral transport

The local dynamics observed above can be harnessed for quantized chiral transport through the Floquet engineering. We show that a topological chiral transport can be achieved by constructing a helical Floquet channel^52^ which leads to a perfect spin-momentum locking through the winding of the Floquet Bloch bands. The helical Floquet channel is the Floquet counterpart of the helical edge states in 2D topological insulators with time-reversal symmetry^53–55^. These states feature directional quantum transport with intrinsic spin-dependent propagation, making them ideal candidates for quantum device design.

**Fig. 3:**
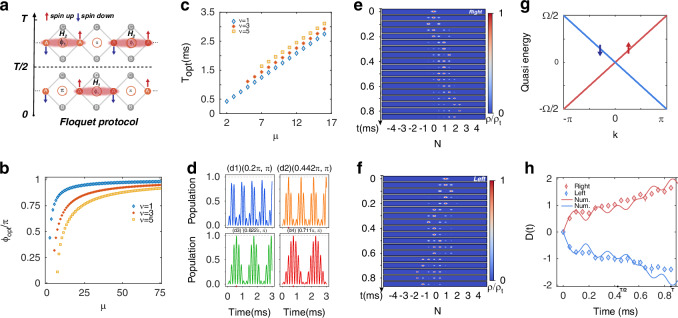
Floquet channel and topological chiral transport. **a** The Floquet protocol by exchanging the adjacent synthetic flux at the spectroscopic moments. In the first half T/2 period, the system can be described by the Hamiltonian *H*_1_; In the second half T/2 period, the system is switched to the Hamiltonian *H*_2_. **b** The theoretical results of optimal flux setting with perfect population transfer. **c** The calculated optimal half Floquet period time *T*_opt_ with perfect transfer corresponding to each *ϕ*_opt_. **d** The transfer dynamics within the spinor pair with different *ϕ*_1_ settings. The subfigure (d1) shows the population transfer under the {*ϕ*_1_ = 0.2*π*, *ϕ*_2_ = *π*} BFL configuration. The last three subfigures (d2–d4) are corresponding to different optimal *ϕ*_1_ = *ϕ*_opt_ settings with *μ*=1, *ν* = 2, 3, 4, respectively (*ϕ*_opt,11_ is an imaginary number and thus is irrelevant). **e**, **f** The experimental results of the Floquet channel with right and left chiral transport, respectively. For the reason that we mainly observe the chiral current represented by the spinor pair sites *A*_*n*_, it is unnecessary to distinguish the micromotions of atomic density in the *B*_*n*_ and *C*_*n*_ sites. So, we have combined the display of *B*_*n*_ and *C*_*n*_ sites by indicating their total density information. **g** The quasi-energy spectrum of the Floquet channel protocol with Ω = 2*π*/*T*. **h** The extracted ${{\mathcal{D}}}(t)$ curve of experimental result for the chiral transports of the Floquet channels. To indicate the chiral feature, we add ± signs in the front of *D*(*t*) to differentiate the leftward and rightward motions. The solid lines show the numerical simulations. The error bars present the standard deviation of measurements

For this purpose, we map the central two sites *A*_2*j*_ and *A*_2*j*+1_ in each unit cell to pseudospins, with the spinor field operator *ψ*_*n*_ = (*ψ*_*n↑*_, *ψ*_*n↓*_) as shown in Fig. 3a. Then, we apply the Floquet protocol by exchanging the neighboring flux to achieve the helical Floquet channel in our BFL configuration. Under this mapping, the chirality of the transport–the propagation directions of atoms are locked with the spin degrees of freedom, is linked to the breaking of the time-reversal symmetry defined by the combination of spin reversal and complex conjugation by preparing different initial states^56^.

Specifically, assuming the wave packet is initialized on a local sublattice site *A*_2*j*_, a perfect population transfer between *A*_2*j*_ and *A*_2*j*+1_ can be achieved under appropriate flux parameters and at a discrete set of times. Analytically, we find that, following an evolution time *t*, the wavefunction on *A*_2*j*_ and *A*_2*j*+1_ are respectively given as $[\cos ({\varepsilon }_{1}t)+\cos ({\varepsilon }_{2}t)]/2$ and $[\cos ({\varepsilon }_{1}t)-\cos ({\varepsilon }_{2}t)]/2$, where *ε*_1_ and *ε*_2_ are the eigen energies of real-space Hamiltonian^46^. It follows that a perfect population transfer occurs at *T*_opt,*μν*_, under the conditions *ε*_1_*T*_opt,*μν*_ = (*μ* + *ν*)*π* and *ε*_2_*T*_opt,*μν*_ = *μπ*, where *μ* is a positive integer and *ν* is a positive odd integer. In terms of the flux parameters, the conditions for the perfect population transfer are given by {*ϕ*_1_ = *ϕ*_opt,*μν*_, *ϕ*_2_ = *π*}, with

2 $${\phi }_{{{\rm{opt}}},\mu \nu }=2\arccos \left[\frac{2\nu (2\mu +\nu )}{{\mu }^{2}+{(\mu +\nu )}^{2}}\right],\,\,\,{{\rm{and}}}\,\,\,\mu \ge \frac{1+\sqrt{3}}{2}\nu$$

In Fig. 3b, we show the discrete values of *ϕ*_opt,*μν*_ that satisfy the conditions above. On the other hand, we also have

3 $${T}_{{{\rm{opt}}},\mu \nu }=\frac{\mu \pi }{J\sqrt{2(2-\cos ({\phi }_{opt,\mu \nu }/2))}}$$

which gives the time of the perfect population transfer, as shown in Fig. 3c.

Note that the above conditions are highly non-trivial, as the full dynamics generically involve the occupation of all the sublattice sites including *B*_*n*_ and *C*_*n*_. Qualitatively, the perfect population transfer derives from the interference between different flat-band components of the initial state.

Based on the flux-dependent local dynamics, we propose to implement a spin-dependent chiral transport as follows. First, we initialize the condensate on-site *A*_*n*_ to the left of the plaquette with flux *ϕ*_opt,*μν*_. We then let the condensate evolve along the momentum lattice, switching the synthetic fluxes *ϕ*_1_ and *ϕ*_2_ within each unit cell at integer multiples of *T*_opt,*μν*_. Taking *ν* = 1 and *μ* = 2 for instance, for a wavefunction initialized on-site *A*_*n*_ under the synthetic-flux configuration {*ϕ*_1_ = *ϕ*_opt,21_ ≈ 0.442*π*, *ϕ*_2_ = *π*}, it propagates to the right and becomes fully localized on-site *A*_*n*+1_ at *t* = *T*_opt,21_ ≈ 0.425 ms when the coupling *J* = *h* × 1.5 kHz. We then swap *ϕ*_1_ and *ϕ*_2_ so that the wavefunction continues to propagate toward the right, becoming fully localized on the central site of the next plaquette (site *A*_*n*+2_) at *t* = 2*T*_opt,21_. By repeating this procedure, a persistent chiral current is realized, which is quantized at discrete time steps. By contrast, the transport is not quantized when, for instance, {*ϕ*_1_ = 0.2*π*, *ϕ*_2_ = *π*}, since the oscillatory dynamics become rather complicated, as illustrated in Fig. 3d.

For the experimental confirmation of the chiral dynamics, we excite both spin-up and spin-down components at *t* = 0, and observe the right- and left-going chiral transport in Fig. 3e, f, respectively. The quantized chiral transport can be understood by neglecting the complicated micromotion, and focusing on coarse-grained Floquet dynamics at discrete time steps *t* = 2*mT*_*opt*,*μν*_ (*m* = 0, 1, 2. . . ). The subsequent dynamics involve only the occupation of the central two sites *A*_*n*_ and *A*_*n*+1_ in each unit cell so that we can map them to pseudospins, with the spinor field operator *ψ*_*n*_ = (*ψ*_*n↑*_, *ψ*_*n↓*_). The stroboscopic Floquet dynamics in the two-dimensional spinor subspace can be described by the Floquet operator $U={e}^{-i{H}_{2}T/2}{e}^{-i{H}_{1}T/2}$, where ${H}_{1}=\frac{\pi }{T}{\sum }_{n}{\psi }_{n}^{{\dagger} }{\sigma }_{x}{\psi }_{n}$, ${H}_{2}=-\frac{\pi }{T}{\sum }_{n}{\psi }_{n}^{{\dagger} }{S}^{+}{\psi }_{n+1}+h.c.$, and ${S}^{+}=\frac{1}{2}({\sigma }_{x}+i{\sigma }_{y})$. Here *T* = 2*T*_opt,*μν*_, and *H*_1_ and *H*_2_ respectively describe the spin-flip process under the switching of flux and the transport process during each half period. Note that coarse-grained effective Hamiltonians are widely used to deal with the stroboscopic dynamics of periodically driven systems^52,57–60^.

In the quasi-momentum space, we have ${U}_{k}(T,0)={e}^{-ik{\sigma }_{z}}$^52^, and it follows that the Floquet Bloch Hamiltonian reads^46^

4 $${H}_{k}^{F}=\frac{i}{T}log[{U}_{k}(T,0)]=\frac{1}{T}k{\sigma }_{z}$$

The Hamiltonian ${H}_{k}^{F}$ has the intrinsic feature of spin-momentum locking in the real space, which underlies the spin-dependent chiral transport. Indeed, this unique feature can be revealed by the Floquet band structure, shown in Fig. 3g, where the quasienergy spectrum is gapless, featuring decoupled linear dispersions for the two spin species. We note that these helical Floquet channels exist at discrete time steps when the interim occupations of sites *B*_*n*_ and *C*_*n*_ are neglected. This is different from previous studies where simpler engineered SSH lattice models were used^61^.

In Fig. 3h, we show the measured chiral transport using the quantity ${{\mathcal{D}}}(t)$. While different spin species flow in different directions, quantized chiral transport is achieved at discrete times *mT*/2 (*m* = 0, 1, 2, . . . ), consistent with the description of the stroboscopic Floquet dynamics. The quantized transport is topologically protected by the Floquet winding numbers^52,57^

5 $${\nu }_{\sigma }=\frac{1}{2\pi i}{\oint }_{BZ}dk{{\rm{Tr}}}[{U}_{k}^{\sigma }{\partial }_{k}{U}_{k}^{\sigma {\dagger} }]$$

where ${U}_{k}^{\uparrow ,\downarrow }={e}^{\pm ik}$ are the irreducible blocks of *U*_*k*_. The Floquet winding numbers reflect the state-dependent winding of quasi-energy bands as the quasi-momentum *k* traverses the Brillouin zone. The winding numbers can also be extracted from the measured ${{\mathcal{D}}}(t)$ in Fig. 3h^46^. They are given by ${\nu }_{\uparrow }={{\mathcal{D}}}(T)/2=0.83\pm 0.05$ (${\nu }_{\downarrow }={{\mathcal{D}}}(T)/2=-0.71\pm 0.05$) for the rightward (leftward) transport, consistent the theoretical predictions *ν*_*↑*_ = 1 (*ν*_*↓*_ = − 1).

## Discussion

We report the induction of topological transport out of a flat-band lattice engineered in the momentum space of ultracold atoms. The underlying helical Floquet channel here are physically richer and technically more demanding than those in ref. ^62^, which features a much simpler dimer chain with no synthetic flux nor flat-band structures. Likewise, our work is also distinct in design and physical mechanism from ref. ^26^, where the disorder-induced delocalization does not lead to a potentially useful transport pattern.

Our approach overcomes the adiabaticity constraints inherent in conventional topological pumping^13^, where adiabatic variation of Hamiltonian parameters is required to achieve quantized charge transport. In contrast, our strategy enables precise modulation of the system’s topological properties without the need to slow its evolution, thereby facilitating robust topological transport even under rapidly changing conditions. This ability to engineer topological transport beyond the adiabatic regime, particularly in a flat-band that facilitates strong correlations, opens new avenues for designing quantum devices aimed at ultrafast transport^63,64^. Moreover, the topological robustness of quantum transport demonstrated here can also be harnessed for applications in quantum metrology^65–68^, such that measurements are not degraded by a broad class of environmental noise or fabrication imperfections.

For the future studies, it would be also interesting to extend the BFL configuration to more complicated structures, including the 2D topological networks^69,70^ and more ingenious lattice configurations for manipulating localized states^71^. The tuning of long-range interactions intrinsic to the momentum-lattice^23,72–74^ would also provide possibilities to study the fate of flat-band localization in the strongly correlated regime^75,76^. Furthermore, we can combine the accessible Feshbach resonances of ^85^Rb or ^133^Cs to tune the inter-atomic interaction strength^77,78^. It would be fascinating to explore the interplay of interaction, flat band, and Floquet engineering for topological transport.

## Materials and Methods

### Experimental implementation

**Fig. 4:**
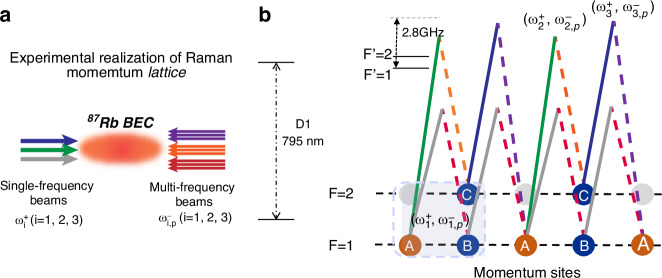
Illustration of experimental configuration and momentum-lattice encoding scheme. **a** The momentum lattice is constructed with three Raman-Bragg laser pairs, which are indicated with different colors and labeled by their corresponding frequencies $\{{\omega }_{j}^{+},{\omega }_{j,p}^{-}\}$ (*j* = 1, 2, 3). **b** Configuration for the multi-frequency Raman-Bragg couplings. The solid (dashed) lines represent the single-frequency (multi-frequency) lasers. The sites *A*_*n*_ and *B*_*n*_ (*C*_*n*_) are encoded with the momentum states of hyperfine level *F* = 1 (*F* = 2). As shown in the figure, the pairs of momentum states $\{\left\vert n,a\right\rangle ,\left\vert n,b\right\rangle \}$ and $\{\left\vert n,b\right\rangle ,\left\vert n+1,a\right\rangle \}$ are all coupled with laser pairs $\{{\omega }_{1}^{+},{\omega }_{1,p}^{-}\}$, while the corresponding momentum states $\{\left\vert n,a\right\rangle ,\left\vert n,c\right\rangle \}$, and $\{\left\vert n,c\right\rangle ,\left\vert n+1,a\right\rangle \}$ are coupled with laser pairs $\{{\omega }_{2}^{+},{\omega }_{2,p}^{-}\}$ and $\{{\omega }_{3}^{+},{\omega }_{3,p}^{-}\}$, respectively. The frequencies ${\omega }_{j,p}^{-}$ are tuned to satisfy the two-photon resonant condition. The single-photon detuning is around 2.8 GHz. The hyperfine split between levels *F* = 1 and *F* = 2 of ^87^*Rb* is about 6.8 GHz

A ^87^Rb Bose-Einstein condensate (BEC) containing approximately 2 × 10^5^ atoms is prepared in a crossed optical dipole trap with trapping frequencies 2*π* × (40, 100, 115) Hz, as shown in Fig. 4a. The lattice model is mapped onto momentum-lattice states with internal hyperfine states as follows: $\left\vert n,a\right\rangle \to \left\vert F=1,p=2n\right\rangle$, $\left\vert n,b\right\rangle \to \left\vert F=1,p=2n+1\right\rangle$, and $\left\vert n,c\right\rangle \to \left\vert F=2,p=2n+1\right\rangle$. Here, *n* denotes the index of the sublattice sites *A*_*n*_, *B*_*n*_, and *C*_*n*_, while *p* represents the real momentum of atoms. The momentum lattice is implemented using Raman-Bragg lasers applied along the weak-trapping axis. Each laser pair consists of a single-frequency beam (solid lines) and multi-frequency beams (dashed lines), with frequencies $\{{\omega }_{j}^{+},{\omega }_{j,p}^{-}\}$ (*j* = 1, 2, 3) coupling adjacent momentum states $\left\vert p\right\rangle$ and $\left\vert p+1\right\rangle$, where *p* is expressed in units of 2*ℏk* (*k* being the wave vector of the 795 nm laser) and ± indicates the propagating directions of beams. As depicted in Fig. 4b, the couplings of lattice sites in the same internal state ({*A*_*n*_, *B*_*n*_} and {*B*_*n*_, *A*_*n*+1_}) are driven by laser pair $\{{\omega }_{1}^{+},{\omega }_{1,p}^{-}\}$^41,42^, while the lattice sites in different internal states ({*A*_*n*_, *C*_*n*_} and {*C*_*n*_, *A*_*n*+1_}) are coupled by the Raman laser pairs of $\{{\omega }_{2}^{+},{\omega }_{2,p}^{-}\}$ and $\{{\omega }_{3}^{+},{\omega }_{3,p}^{-}\}$^26^. The multi-frequency components *ω*_*j*,*p*_ are finely tuned to minimize two-photon detuning.

Following Raman-Bragg coupling, the atomic population distribution is measured via absorption imaging. The probe light addresses atoms in the *F* = 2 level, enabling site-selective detection. For imaging, both the optical dipole trap and Raman-Bragg lasers are switched off, and the atoms undergo a 20 ms time-of-flight expansion. During this period, atoms with different momenta spatially separate, allowing direct extraction of populations in each momentum state. This imaging method provides a clear visualization of the atomic distribution across lattice sites and serves as a key tool for probing the effects of Raman-Bragg coupling in the experiment.

As shown in the energy-level diagram in Fig. 4b, only the *C*_*n*_ sites in the lattice model are encoded in the *F* = 2 state. To determine the population in the individual lattice sites *A*_*n*_, *B*_*n*_ and *C*_*n*_, we employ a two-step measurement process. In the first step, a pumping light transfers the population of each momentum-lattice site in the *F* = 1 state to the *F* = 2 state. An absorption image of the *F* = 2 state then provides the combined population of both internal states for each momentum state. In the second step, we omit the pumping process and take an absorption image directly of the *F* = 2 state, thereby measuring only the population in the *C*_*n*_ sites. If resolving the population of the *F* = 2 state across specific lattice sites is unnecessary, the first step alone suffices, which directly yields the total population of each momentum-lattice site as illustrated in Fig. 3 of the main text. This two-step protocol offers flexibility, enabling targeted characterization of population distributions across different internal states and lattice sites according to experimental needs.

## Supplementary information

Supplemental Material for “Engineering topological chiral transport in a flat-band lattice of ultracold atoms”

## Data Availability

All the data supporting the findings of this study are available within this article and its dmmc1Supplementary Information. Any additional information can be obtained from corresponding authors on reasonable request.
