# Supplementary material for: Engineering topological chiral transport in a flat-band lattice of ultracold atoms
Source: Light Sci Appl. 2025 Sep 17;14:326. doi: 10.1038/s41377-025-02025-3 (PMC12441146; doi:10.1038/s41377-025-02025-3)
Supplement: Supplementary file 1 — Supplemental Material for “Engineering topological chiral transport in a flat-band lattice of ultracold atoms” [file 41377_2025_2025_MOESM1_ESM.pdf]

Supplemental Material for "Engineering topological chiral transport in a flat-band lattice of ultracold atoms"

Hang Li,[1,†], Qian Liang,[1,†], Zhaoli Dong[1], Hongru Wang[1], Wei Yi[2,3,4], Jian-Song Pan,[5*] Bo Yan,[1*]

[1]Zhejiang Province Key Laboratory of Quantum Technology and Device, School of Physics, and State Key Laboratory for Extreme Photonics and Instrumentation, Zhejiang University, Hangzhou 310027, China.

[2]Laboratory of Quantum Information, University of Science and Technology of China, Hefei 230026, China,

[3]Anhui Province Key Laboratory of Quantum Network, University of Science and Technology of China, Hefei 230026, China,

[4]CAS Center For Excellence in Quantum Information and Quantum Physics, Hefei 230026, China,

[5]College of Physics and Key Laboratory of High Energy Density Physics, and Technology of Ministry of Education, Sichuan University, Chengdu 610065, China,

[†]These authors contributed equally to this work.

[*]email: panjsong@scu.edu.cn

[*]email: yanbohang@zju.edu.cn

This Supplementary Information consists of the following sections:

I. Experimental details

II. Derive the effective Hamiltonian

III. Energy bands of static flat-band lattice

IV. Simulating the initial state preparation and biased oscillation

V. Detecting the Chiral edge states

VI. Optimal engineering scheme for chiral transport

VII. Equivalence between winding numbers and transport distances of condensate

I. Experimental details

Here, we give some details about the mapping scheme of lattice model in the main text. The relationship of lattice model and momentum-lattice states with internal hyperfine states can be expressed as

$$|n,a\rangle \rightarrow |F=1,p=2n\rangle \tag{S1}$$
$$|n,b\rangle \rightarrow |F=1,p=2n+1\rangle \tag{S2}$$
$$|n,c\rangle \rightarrow |F=2,p=2n+1\rangle \tag{S3}$$

Here, n stands for the index of the n_{th} sublattice sites A_n, B_n, and C_n, and p in the right side represents real momentum. As the Figure 4 of the main text shows, the laser frequencies are denoted as $\{\omega_j^+, \omega_{j,p}^-\}$ ($j = 1,2,3$), and they couple adjacent momentum states $|p\rangle$ and $|p+1\rangle$ (where p denotes momentum states in units of $2\hbar k$, with k being the wave vector of the 795 nm laser, and $\pm$ indicating the propagating direction of the beams). The bonding lattice sites encoded with the same internal state, $\{A_n, B_n\}$ and $\{B_n, A_{n+1}\}$, are coupled with the Raman-Bragg laser pairs $\{\omega_1^+, \omega_{1,p}^-\}$ (*1, 2*), while for the bonding lattice sites encoded with different internal states, $\{A_n, C_n\}$ sites and $\{C_n, A_{n+1}\}$, are coupled with the other two laser pairs $\{\omega_2^+, \omega_{2,p}^-\}$ and $\{\omega_3^+, \omega_{3,p}^-\}$ (*3*), respectively.

Concretely, the lasers with frequencies $\{\omega_1^+, \omega_{1,p}^-\}$ couple momentum states in the same internal state satisfy (*3, 4*),

$$|n,a\rangle \leftrightarrow |n,b\rangle \ \text{ with } \ \omega_{1,p}^- = \omega_1^+ - (4n+1)4E_r/\hbar, \tag{S4}$$
$$|n,b\rangle \leftrightarrow |n+1,a\rangle \ \text{ with } \ \omega_{1,p}^- = \omega_1^+ - (4n+3)4E_r/\hbar. \tag{S5}$$

The lasers with frequencies $\{\omega_2^+, \omega_{2,p}^-\}$ couple $|n,a\rangle \leftrightarrow |n,c\rangle$, in the two-photon processes take the frequencies

$$\omega_{2,p}^- = \omega_2^+ - \Delta_{\text{hyp}} - (4n+1)4E_r/\hbar. \tag{S6}$$

Here $E_r = \hbar^2 k^2/2M$ is the recoil energy, and $\Delta_{\text{hyp}} \approx 6.8GHz$ is the hyperfine splitting between the ground state manifolds $F=1$, $F=2$. Similarly, the lasers with $\{\omega_3^+, \omega_{3,p}^-\}$ couple $|n,c\rangle \leftrightarrow |n+1,a\rangle$, in the two-photon processes take the frequencies

$$\omega_{3,p}^- = \omega_3^+ + \Delta_{\text{hyp}} - (4n+3)4E_r/\hbar. \tag{S7}$$

The dynamics of BEC on the synthetic momentum lattice elaborated here can be characterized by the effective Hamiltonian in Eq. (1) of the main text.

In our system, the coupling coefficients of the lattice model need to be calibrated before the data acquisition. The two typical Rabi couplings in this work are shown in Fig. S1. From the coefficients calibration, we evaluate the decoherence time of our system to be around 1 ms.

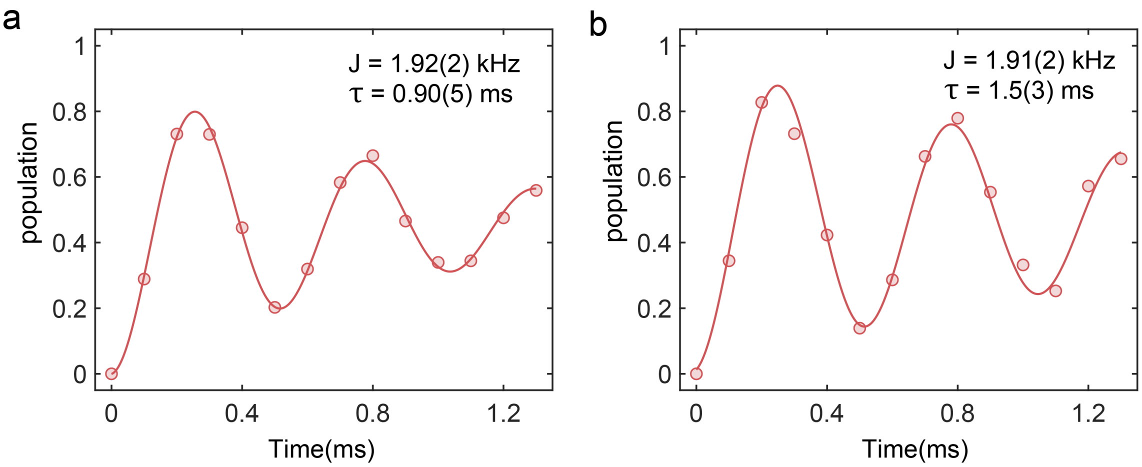

Figure S1: **Characterizing the Rabi frequency. a.** The calibrated Rabi frequency in the F=1 internal state, here the Rabi frequency is twice the coupling strength of the Hamiltonian. The fitted decoherence lifetime is around 1 ms. **b.** The calibrated Rabi frequency between the F=1 and F=2 internal state, and the fitted decoherence lifetime is about 1.5 ms.

The results of Fig. S1 show that the Rabi oscillations can be maintained relatively well even after 0.6 ms. The overall impact of decoherent effect is the apparent deviation of the dynamics from that driven by the effective Hamiltonian (1) when the evolution time is ~ 1 ms. The decoherence time shown by the Rabi oscillation in Fig. S1 is ~ 1 ms, while the decoherence time extracted from Fig. 1(e) is $\sim 0.50(5)$ ms (the time for preparing initial state should be taken into consideration). The main reason behind this difference is that the actual experiments shown in the figures of the main text contain many pairs of Raman-Bragg lasers, which may enhance the decoherence effect in a relatively long momentum-lattice chain.

In general, decoherence does not give rise to significant heating or atom loss. It does lead to a localization of the wave packet, due to the decoherence between different hopping channels. The dynamic evolution in a momentum lattice is mainly limited by the decoherence time. In our set-up, decoherence comes from several sources. First, the inhomogeneous density distribution of BEC in the trapping potential can lead to inhomogeneous interactions in the momentum space, giving rise to decoherence. As a feasible way to improve it, one can weaken the global trapping potential. Second, the phase noise of lasers can also lead to decoherence [see relevant discussions in reference (*5*)]. In principle, further mitigation of decoherence is possible, which can be realized by instantaneous phase locking or by filtering the phase noise by an ultrastable cavity. Nevertheless, we would like to emphasize that the decoherence lifetime of our momentum lattice is on par with the current state of the art (*6, 7, 8*).

II. Derive the effective Hamiltonian

The optical field of the Raman momentum lattice can be expressed as:

$$\boldsymbol{E} = \sum_j E_j^+ \cos\left(k_j^+ \hat{x} - \omega_j^+ t + \phi_j^+\right) + \sum_{i,j} E_{i,j}^- \cos\left(k_{i,j}^- \hat{x} - \omega_{i,j}^- t + \phi_{i,j}^-\right) \tag{S8}$$

Here, i is the multi-frequency index for the Raman-Bragg light, and $j = 1, 2, 3$ represents the three transitions described earlier. The Hamiltonian can be written as:

$$\begin{aligned}\hat{H} = &\frac{\hat{p}^2}{2M} + \hbar\omega_{\mathrm{e}}|e\rangle\langle e| + \hbar\omega_{\mathrm{g}_1}|g_1\rangle\langle g_1| + \hbar\omega_{\mathrm{g}_2}|g_2\rangle\langle g_2| \\ &+ \frac{\hbar}{2}\sum_{l=1,2}\left[\left(\sum_j \Omega_j^{+,l} e^{i\left(k\hat{x}-\omega_j^+ t+\phi_j^+\right)} + \sum_{i,j}\Omega_{i,j}^{-,l} e^{i\left(-k\hat{x}-\omega_{i,j}^- t+\phi_{i,j}^-\right)}\right)|e\rangle\langle g_l| + \text{ h.c.}\right]\end{aligned} \tag{S9}$$

To simplify the notation, $l = 1, 2$ is used as the index for the ground states g_1 and g_2 (F=1 and F=2 in Fig.S1(b)). The quantities $\Omega_j^{+,l}$ and $\Omega_{i,j}^{-,l}$ are defined as the single-photon Rabi frequencies for transitions from the two ground states to the excited state. In the interaction picture, the momentum-space wave function is:

$$|\psi\rangle = \int dp\left[\sum_{l=1,2}\alpha_{g_l}(p)e^{-i\left(p^2/2M+\hbar\omega_{g_l}\right)t/\hbar}|g,p\rangle + \beta(p)e^{-i\left(p^2/2M+\hbar\omega_e\right)t/\hbar}|e,p\rangle\right] \tag{S10}$$

Here, we express the wave functions under the momentum-space picture with the momentum of atoms $p = 2n\hbar k$, n is the quantum number of momentum states. Notably, $e^{\pm ik\hat{x}}$ are the ladder operators for momentum states with the relationship of $e^{\pm ik\hat{x}} = \int dp\,|p \pm \hbar k\rangle\,\langle p|$.

Substituting into the Schrödinger equation and defining $\omega_{eg_l} = \omega_e - \omega_{g_l}$, we obtain the follow equations in the interaction picture:

$$\begin{aligned} i\partial_t\alpha_{g_l}(p) = &\sum_j \frac{\Omega_j^{+,l*}}{2} e^{i[(\omega_j^+ - \omega_{eg_l})t - \phi_j^+]} e^{-i(p\hbar k/M + E_r)t/\hbar}\beta(p+\hbar k) \\ &+ \sum_{i,j}\frac{\Omega_{i,j}^{-,l*}}{2} e^{i[(\omega_{i,j}^- - \omega_{eg_l})t - \phi_{i,j}^-]} e^{i(p\hbar k/M - E_r)t/\hbar}\beta(p-\hbar k) \end{aligned} \tag{S11}$$

$$\begin{aligned} i\partial_t\beta(p) = \sum_{l=1,2}\Bigg[&\frac{\Omega_j^{+,l}}{2} e^{i[(-\omega_j^+ + \omega_{eg_l})t - \phi_j^+]} e^{i(p\hbar k/M - E_r)t/\hbar}\alpha_{g_l}(p-\hbar k) \\ &+ \sum_{i,j}\frac{\Omega_{i,j}^{-,l}}{2} e^{i[(-\omega_{i,j}^- + \omega_{eg_l})t - \phi_{i,j}^-]} e^{-i(p\hbar k/M + E_r)t/\hbar}\alpha_{g_l}(p+\hbar k)\Bigg] \end{aligned} \tag{S12}$$

In the experiment, the single-photon detuning is on the order of GHz, much larger than the frequency difference of the Raman-Bragg light. Thus, the single-photon detuning can be defined as $\Delta_i^l = \omega_i^+ - \omega_{eg_l} \approx \omega_{i,j}^- - \omega_{eg_l}$. Integrating Eq. (S12), we obtain:

$$\beta(p) \approx \sum_{l=1,2} \left[\sum_j \frac{\Omega_j^{+,l}}{2\Delta_j^l} e^{-i[(-\omega_j^+ + \omega_{eg_l})t + \phi_j^+]} e^{i(p\hbar k/M - E_r)t/\hbar} \alpha_{gl}(p - \hbar k) + \sum_{i,j} \frac{\Omega_{i,j}^{-,l}}{2\Delta_j^l} e^{-i[(-\omega_{i,j}^- + \omega_{eg_l}) - \phi_j^-]} e^{-i(p\hbar k/M + E_r)t/\hbar} \alpha_{gl}(p + \hbar k) \right] \tag{S13}$$

where E_r is the recoil energy defined in the last section. Substituting (S13) into (S11) and adiabatically eliminating the excited state, the population dynamics of the two ground states are obtained:

$$\begin{aligned} i\partial_t \alpha_{g_l}(p) = \sum_{l'=1,2} e^{i(\omega_{egl'} - \omega_{egl})} \Bigg[&\sum_{j,j'} \frac{\Omega_{j'}^{+,l'} \Omega_j^{+,l*}}{4\Delta_{j'}^{l'}} e^{i\left[\left(\omega_j^+ - \omega_{j'}^+\right)t - \left(\phi_j^+ - \phi_{j'}^+\right)\right]} \alpha_{g_{l'}}(p) \\ &+ \sum_{i,i',j,j'} \frac{\Omega_{i',j'}^{-,l'} \Omega_{i,j}^{-,l*}}{4\Delta_{j'}^{l'}} e^{i\left[\left(\omega_{i,j}^- - \omega_{i',j'}^-\right)t - \left(\phi_{i,j}^- - \phi_{i',j'}^-\right)\right]} \alpha_{g_{l'}}(p) \\ &+ \sum_{i,j,j'} \frac{\Omega_{j'}^{+,l'} \Omega_{i,j}^{-,l*}}{4\Delta_{j'}^{l'}} e^{i\left[\left(\omega_{i,j}^- - \omega_{j'}^+\right)t - \left(\phi_{i,j}^- - \phi_{j'}^+\right)\right]} e^{i(2p\hbar k/M - 4E_r)t/\hbar} \alpha_{g_{l'}}(p - 2\hbar k) \\ &+ \sum_{i',j,j'} \frac{\Omega_{i',j'}^{-,l'} \Omega_j^{+,l*}}{4\Delta_{j'}^{l'}} e^{i\left[\left(\omega_j^+ - \omega_{i',j'}^-\right)t - \left(\phi_j^+ - \phi_{i',j'}^-\right)\right]} e^{-i(2p\hbar k/M + 4E_r)t/\hbar} \alpha_{g_{l'}}(p + 2\hbar k) \Bigg] \end{aligned} \tag{S14}$$

Using the integer n as the quantum number for the momentum state, $p = 2n\hbar k$. With $|g_l, n\rangle$

as the basis, the full Hamiltonian of the system can be written as:

$$\hat{H}_{\text{full}} = \hbar \sum_n \Bigg\{ \sum_{l=1,2} \Bigg[\sum_{j,j'} \frac{\Omega^{+,l}_{j'} \Omega^{+,l*}_{j}}{4\Delta^l_{j'}} e^{i\left[\left(\omega^+_j - \omega^+_{j'}\right)t - \left(\phi^+_j - \phi^+_{j'}\right)\right]}$$

$$+ \sum_{i,i',j,j'} \frac{\Omega^{-,l}_{i',j'} \Omega^{-,l*}_{i,j}}{4\Delta^l_{j'}} e^{i\left[\left(\omega^-_{i,j} - \omega^-_{i',j'}\right)t - \left(\phi^-_{i,j} - \phi^-_{i',j'}\right)\right]} \Bigg] |g_l, n\rangle \langle g_l, n| \tag{S15}$$

$$+ \Bigg[\sum_{j,j'} \frac{\Omega^{+,1}_{j'} \Omega^{+,2*}_{j}}{4\Delta^1_{j'}} e^{i\left[\left(\omega^+_j - \omega^+_{j'} + \Delta_{\text{hyp}}\right)t - \left(\phi^+_j - \phi^+_{j'}\right)\right]}$$

$$+ \sum_{i,i',j,j'} \frac{\Omega^{-,1}_{i',j'} \Omega^{-,2*}_{i,j}}{4\Delta^1_{j'}} e^{i\left[\left(\omega^-_{i,j} - \omega^-_{i',j'} + \Delta_{\text{hyp}}\right)t - \left(\phi^-_{i,j} - \phi^-_{i',j'}\right)\right]} \Bigg] |g_2, n\rangle \langle g_1, n| \tag{S16}$$

$$+ \sum_{l=1,2} \sum_{i,j,j'} \frac{\Omega^{+,l}_{j'} \Omega^{-,l*}_{i,j}}{4\Delta^l_{j'}} e^{i\left[\left(\omega^-_{i,j} - \omega^+_{j'}\right)t - \left(\phi^-_{i,j} - \phi^+_{j'}\right)\right]} e^{i4(2n+1)E_r t/\hbar} |g_l, n+1\rangle \langle g_l, n| \tag{S17}$$

$$+ \sum_{i,j,j'} \frac{\Omega^{+,1}_{j'} \Omega^{-,2*}_{i,j}}{4\Delta^1_{j'}} e^{i\left[\left(\omega^-_{i,j} - \omega^+_{j'} + \Delta_{\text{hyp}}\right)t - \left(\phi^-_{i,j} - \phi^+_{j'}\right)\right]} e^{i4(2n+1)E_r t/\hbar} |g_2, n+1\rangle \langle g_1, n|$$

$$+ \sum_{i,j,j'} \frac{\Omega^{+,2}_{j'} \Omega^{-,1*}_{i,j}}{4\Delta^1_{j'}} e^{i\left[\left(\omega^-_{i,j} - \omega^+_{j'} - \Delta_{\text{hyp}}\right)t - \left(\phi^-_{i,j} - \phi^+_{j'}\right)\right]} e^{i4(2n+1)E_r t/\hbar} |g_1, n+1\rangle \langle g_2, n| + \text{h.c.} \Bigg\} \tag{S18}$$

The full Hamiltonian consists of four parts. The first term (S15) represents the light shift. To avoid off-resonant coupling, we adjust ω^+_j in the experiment such that $\omega^+_j - \omega^+_{j'} \sim$ MHz, $(j \neq j')$. Thus, all terms with $j \neq j'$ are rapidly oscillating and can be neglected. For all $i = i'$, this term represents a time-independent energy shift between the two internal states, which is equal for all momentum states. In the experiment, this shift can be compensated by setting the two-photon detuning of the Raman light. For $i \neq i'$, the term represents a time-dependent light shift, which is negligible when the single-photon Rabi frequency $\Omega^-_{i,j}$ is small.

The second term (S16) represents Raman coupling without momentum transfer. In the frequency configuration of the AB cage, there is no Raman coupling, so this term is rapidly oscillating and can be neglected.

The third term (S17) represents Bragg coupling within the same internal state. According to the configuration in Fig.4(b) of main text, only terms with indices $j = j' = 3$, $i = n$ are resonant, while all others are detuned and can be neglected. Note that in the ideal AB cage structure, Bragg coupling exists only for the ground state g_1. However, (S17) shows that Bragg coupling also exists for g_2, with different tunneling strengths $\frac{\Omega^{+,l}_{j'} \Omega^{-,l*}_{i,j}}{4\Delta^l_{j'}}$ for the two internal

states, but identical tunneling phases and two-photon resonance conditions. In the experiment, the single-photon detunings are set to $\Delta_3^1 = 2.8$ GHz and $\Delta_3^2 = 9.6$ GHz. Here, $\frac{\Omega_3^{+,2}\Omega_{i,3}^{-,2*}}{4\Delta_3^2} \approx 0.1 \times \frac{\Omega_3^{+,1}\Omega_{i,3}^{-,1*}}{4\Delta_3^1}$. In a rough treatment, the coupling for g_2 can be ignored (*4*). The tunneling strength between sites A_n and B_n is defined as $\frac{\Omega_3^{+,1}\Omega_{2n,3}^{-,1*}}{4\Delta_3^1} = J_{ab}/\hbar$, with tunneling phase θ_{ab}. The tunneling strength between sites B_n and A_{n+1} is $\frac{\Omega_3^{+,1}\Omega_{2n+1,3}^{-,1*}}{4\Delta_3^1} = J_{ba}/\hbar$, with tunneling phase θ_{ba}.

The final term (S18) represents Raman-Bragg coupling. After neglecting off-resonant terms, the tunneling strength between sites A_n and C_n is defined as $\frac{\Omega_1^{+,1}\Omega_{2n,3}^{-,2*}}{4\Delta_1^1} = J_{ac}/\hbar$, with tunneling phase θ_{AC}. The tunneling strength between sites C_n and A_{n+1} is $\frac{\Omega_2^{+,2}\Omega_{2n+1,2}^{-,1*}}{4\Delta_2^2} = J_{ca}/\hbar$, with tunneling phase θ_{ca}.

Finally, we obtain the effective Hamiltonian of the diamond chain in Fig.4(b) of main text:

$$\begin{aligned} H_{\text{eff}} = \sum_n \Big[& J_n^{ba} e^{i\theta_n^{ba}} |A, n+1\rangle\langle B, n| + J_n^{ab} e^{i\theta_n^{ab}} |B, n\rangle\langle A, n| + J_n^{ca} e^{i\theta_n^{ca}} |A, n+1\rangle\langle C, n| \\ & + J_n^{ac} e^{i\theta_n^{ac}} |C, n\rangle\langle A, n| + \text{ h.c. } \Big] \end{aligned} \tag{S19}$$

III. Energy bands of static flat-band lattice

From the effective Hamiltonian in the main text [Eq. (1)], we can easily write the quasimomentum Hamiltonian in the BFL configuration, which reads as

$$H_{\text{Bulk}} = J \begin{bmatrix} 0 & 1 & 1 & 0 & e^{-i2ka} & e^{-i(2ka+\phi_2)} \\ 1 & 0 & 0 & 1 & 0 & 0 \\ 1 & 0 & 0 & e^{i(\phi_1)} & 0 & 0 \\ 0 & 1 & e^{-i(\phi_1)} & 0 & 1 & 1 \\ e^{i2ka} & 0 & 0 & 1 & 0 & 0 \\ e^{i(2ka+\phi_2)} & 0 & 0 & 1 & 0 & 0 \end{bmatrix}, \tag{S20}$$

where we have taken the artificial gauge defined as the main text does, and we can also set the lattice constant $a = 1$ for convenience. Through diagonalizing the bulk Hamiltonian, we can get the dispersion-less eigenvalues as

$$\begin{aligned} E_0 &= 0, \\ E_{\pm 2} &= \pm J\sqrt{4 + \sqrt{2F(\phi_1, \phi_2)}}, \\ E_{\pm 1} &= \pm J\sqrt{4 - \sqrt{2F(\phi_1, \phi_2)}} \end{aligned} \tag{S21}$$

where $F(\phi_1, \phi_2) = 2 + \cos(\phi_1) + \cos(\phi_2) + 4\cos(2ka)\cos(\phi_1/2)\cos(\phi_2/2)$, and k $(-\pi \leq k < \pi)$ is the Bloch wavenumber. So, we can get the dispersion relationships of the $\{\phi_2 = \pi\}$

BFL case in the Fig. 1(c) of main text as $E_0 = 0$, $E_{\pm 1} = \pm J\sqrt{4 - 2\cos(\phi_1/2)}$, and $E_{\pm 2} = \pm J\sqrt{4 + 2\cos(\phi_1/2)}$ with the flux-dependent factor $F(\phi_1, \phi_2) = 2\cos^2(\phi_1/2)$.

Under the BFL configurations with $\phi_1 = 0$ and $\phi_2 = \pi$, we have experimentally confirm the biased oscillation in the main text. In this case, the eigenvalues of system are $E_0 = 0$, $E_{\pm 1} = \pm J\sqrt{2}$, and $E_{\pm 2} = \pm J\sqrt{6}$, where the E_0 is two-fold degenerate. The corresbonding eigenstates has the forms as follows

$$\left|\psi_{n,\pm 2}\right\rangle = \frac{1}{2}[(\hat{a}^{\dagger}_{n+1} + \hat{a}^{\dagger}_{n}) \pm \frac{\sqrt{6}}{6}(2\hat{b}^{\dagger}_{n} + 2\hat{c}^{\dagger}_{n} - \hat{b}^{\dagger}_{n-1} + \hat{c}^{\dagger}_{n-1} + \hat{b}^{\dagger}_{n+1} + \hat{c}^{\dagger}_{n+1})] \left|0\right\rangle \tag{S22}$$

$$\left|\psi_{n,\pm 1}\right\rangle = \frac{1}{2}[(\hat{a}^{\dagger}_{n+1} - \hat{a}^{\dagger}_{n}) \pm \frac{\sqrt{2}}{2}(\hat{b}^{\dagger}_{n-1} - \hat{c}^{\dagger}_{n-1} + \hat{b}^{\dagger}_{n+1} + \hat{c}^{\dagger}_{n+1})] \left|0\right\rangle \tag{S23}$$

$$\left|\psi_{n,\,0}\right\rangle = \frac{\sqrt{2}}{2}(\hat{b}^{\dagger}_{n} - \hat{c}^{\dagger}_{n}) \left|0\right\rangle \tag{S24}$$

The initial state used in the Fig. 1(d) of main text, defined as $|\psi_{\text{ini}}\rangle = \frac{1}{\sqrt{2}}\left(\hat{a}^{\dagger}_{n} + \hat{a}^{\dagger}_{n+1}\right)|0\rangle$, represents an equal-weight superposition of the two basis states $|\psi_{n,+2}\rangle$ and $|\psi_{n,-2}\rangle$. This can equivalently be expressed as the projective form:

$$|\psi_{\text{ini}}\rangle = \frac{1}{\sqrt{2}}\left(|\psi_{n,+2}\rangle + |\psi_{n,-2}\rangle\right),$$

where the subscript indices ± 2 correspond to distinct energy bands $E_{\pm 2}$. Consequently, the characteristic breathing-mode dynamics observed in this system arise from the energy difference (gap) between the E_{+2} and E_{-2} bands, resulting in an oscillation frequency proportional to $\Delta E = |E_{+2} - E_{-2}|$.

IV. Simulating the initial state preparation and biased oscillation

In the observation of the breathing mode illustrated in Fig. 1(c) of the main text, the preparation of the initial state $|\psi_{ini}\rangle = \frac{1}{\sqrt{2}}(a^{\dagger}_{n} + a^{\dagger}_{n+1})|0\rangle$ involves splitting the initial BEC wave packet at site B of the middle unit cell (labeled as 0_{th}). The process is detailed in Fig. S2(a). By encoding the appropriate hopping rates J_{c1}, J_{c2}, hopping time of τ, and hopping phases of $\varphi_i (i = c1, c2)$ for each coupling, the initial state can be generated with designed amplitudes and relative phases.

To achieve this symmetry state in our experimental setup, specific values for the hopping rates and phases were chosen. In particular, we set $J_{c1} = J_{c2} = 3.50(2)$ kHz with the hopping phases of $\varphi_{c1} = \pi/2$, $\varphi_{c2} = 3\pi/2$. After an evolution time of 0.1 ms, the prepared initial state $|\psi_{ini}\rangle$ is realized, as depicted in Fig. S2(b). This controlled preparation of the initial state is crucial for the subsequent observation of the breathing mode in the system.

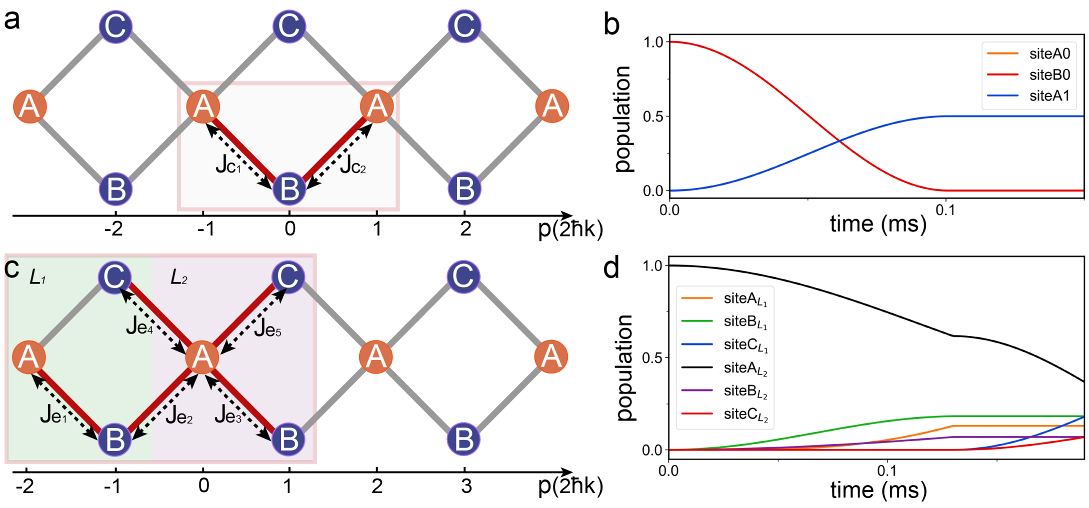

Figure S2: **The preparation of the initial state and its evolutions**. **a**, The preparation protocol of the initial state. The experimental parameters are $J_{c1} = J_{c2} = 3.50(2)$ kHz, and the corresponding hopping phases are $\varphi_{c1} = \pi/2$, $\varphi_{c2} = 3\pi/2$. **b**, During a 0.1 ms interval, the initial BEC is evolved to the desired state. The population evolutions of the initial Gaussian state on the four lattice sites. Note that population evolution on site A_0 overlaps with that on site A_1. **c**, The preparation protocol of the initial edge state. There are two steps to generate this edge state. In the first step, we use three appropriate hopping rates of J_{e1}=3.30(3) kHz, J_{e2}=1.60(2) kHz, J_{e3}=0.70(3) kHz with the hopping phases of $\varphi_{e1} = \pi/2$, $\varphi_{e2} = \pi/2$, $\varphi_{e3} = -\pi/2$, respectively. We then prepare the initial four-site state through a time evolution of τ_1= 0.1 ms. Then, we turn off the couplings for the first step, and switch on the hooping process characterized by J_{e4}=3.10(3) kHz, J_{e5}=1.90(2) kHz with the hopping phases of $\varphi_{e4} = \pi/2$, $\varphi_{e5} = -\pi/2$, respectively. After a time evolution of 0.1 ms in the second step, we get a six-site edge state. **d**, The population evolution of different sites during the two steps in **c**.

Figure S3 presents the numerical simulation results of the localized biased oscillation dynamics, as discussed in Fig. 2 of the main text. These simulations are derived from the effective Hamiltonian (H_{eff}). Upon comparison with the experimental results shown in Fig. 2, it becomes evident that decoherence delivers a strong impact on the evolution dynamics after 0.6 ms. The discrepancy between the simulated and experimental results highlights the influence of the decoherence effect on the observed dynamics.

In Fig. S4, we show both $\mathcal{D}(t)$ and the average positions calculated from our experimental data. Here the average position of the center of mass is defined as $p(t) = \sum_n (n - n_0)(|a_n|^2 + |b_n|^2 + |c_n|^2)$, where n_0 is the initial condensate location. While both parameters provide qualitatively consistent characterizations of the dynamic features, we opt to choose $\mathcal{D}(t)$ over the average position for the following reasons.

First, the $\mathcal{D}(t)$ curve shows more apparent oscillatory features, theoretically characterized

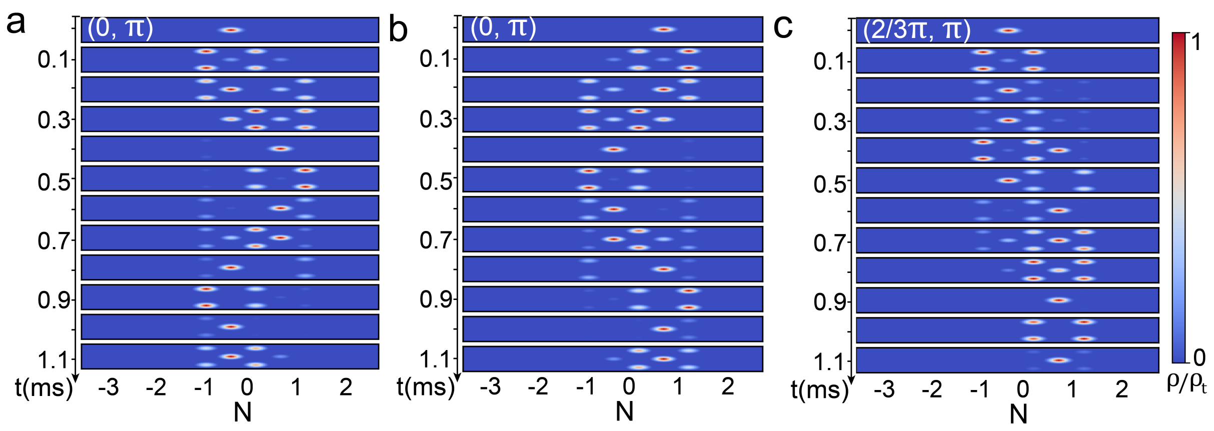

Figure S3: **Simulating the biased oscillation in the BFL configuration. a.** The numerical dynamics under $\{\phi_1 = 0, \phi_2 = \pi\}$, with the initial state $|\psi_{\rm ini}\rangle = \hat{a}_0^\dagger|0\rangle$.. **b.** The numerical dynamics under $\{\phi_1 = 0, , \phi_2 = \pi\}$ with $|\psi_{\rm ini}\rangle = \hat{a}_1^\dagger|0\rangle$. **c.** The numerical dynamics under $\{\phi_1 = 2\pi/3, , \phi_2 = \pi\}$ with $|\psi_{\rm ini}\rangle = \hat{a}_0^\dagger|0\rangle$.

by two oscillating frequencies of $E_2 + E_1 = (\sqrt{4 + 2\cos(\phi_1)} + \sqrt{4 - 2\cos(\phi_1)})J$ and $E_2 - E_1 = (\sqrt{4 + 2\cos(\phi_1)} - \sqrt{4 - 2\cos(\phi_1)})J$. We extract the frequencies from the experimental data by fitting the measured $\mathcal{D}(t)$ using the function $d(t) = a_1 \sin[(\omega_2+\omega_1)t+\phi_1]exp(-t/\tau_1)+ a_2 \sin[(\omega_2 - \omega_1)t + \phi_2] \exp(-t/\tau_2) + c$. The fitting results of Fig. S4(a) give ω_1 =1.4(1) kHz, ω_2 =2.6(1) kHz for the $(0, \pi)$ case, and ω_1 =1.7(1) kHz, ω_2 =2.3(1) kHz for the $(2\pi/3, \pi)$ case, respectively. For comparison, the theoretically predicted values are ω_1 =1.42 kHz, ω_2 =2.45 kHz for the $(0, \pi)$ case and ω_1 =1.73 kHz, ω_2 =2.23 kHz for the $(2\pi/3, \pi)$ case, respectively. However, it is difficult to fit the exact oscillation frequency for the average position curve in Fig. S4, where the oscillatory behaviors are smoothed out at intermediate moments. From this perspective, we believe that the $\mathcal{D}(t)$ curve can better reflect the characteristic biased oscillatory dynamics, which is consistent with the energy spectrum of Fig. 1(c) of the main text.

Second, the $\mathcal{D}(t)$ operator is relatively less sensitive to the effect of decoherence. As shown in Fig. S4(b), the measured average positions show a larger deviation from the theoretical prediction after 0.6 ms.

V. Detecting the Chiral edge states

As an example to demonstrate the flexibility of our binary-flux staggered momentum lattice and possible interesting extensions of our model, we add an experimental study of edge modes under the open boundary condition. Accompanied by the biased localization dynamics shown in the bulk states of BFL configuration, there are also chiral edge states appearing at only one edge of the chain. This is understandable as the intrinsic reflection-symmetry breaking of the

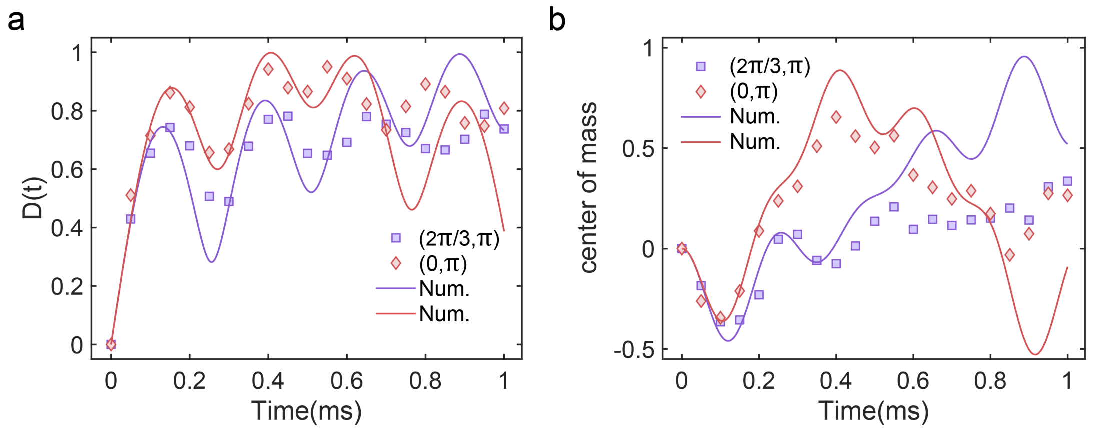

Figure S4: **Simulating the biased oscillations with different parameters. a.** The extracted $\mathcal{D}(t)$ curve of simulation for 4 cases of flux setting. **b.** The extracted center-of-mass curve of simulation for 4 cases of flux setting.

staggered-flux structure. As shown in Fig. S5(a), the quasienergy spectrum has four chiral edge states at the left edge of the BFL. We take the top left-chiral edge state (highlighted in red in Fig. S5(a)) for instance, which is given by $\left|\psi_{\text{top}}^{\text{edge}}\right\rangle = (0.372a_{L_1}^\dagger + 0.425b_{L_1}^\dagger + 0.425c_{L_1}^\dagger + 0.602a_{L_2}^\dagger + 0.263b_{L_2}^\dagger + 0.263c_{L_2}^\dagger)\left|0\right\rangle$, populating in the left-most L_1 and L_2 unit cells. In the experiment, we prepare the exact form of this left chiral edge state to observe its localization, as shown in Fig. S5(b). As the control group, we also prepare the same state but at the right edge of BFL, and observe its evolution. The delocalization behavior is displayed in Fig. S5(c). To distinguish the above localization and delocalization evolutions, we show their respective displacement $\mathcal{D}(t)$ in Fig. S5(d). The state $\left|\psi_{\text{top}}^{\text{edge}}\right\rangle$ localizes well at the left edge while evolving into the bulk at the right edge.

For the initial-state preparation here (see Fig. S2(c) and (d)), we need to compensate for the extra cumulative phases brought about by the light shift in different hyperfine manifolds to ensure correct relative phases between wave-function components on different momentum-lattice sites.

VI. Optimal engineering scheme for chiral transport

In this subsection, the details underlying the optimal Floquet engineering scheme for chiral current are presented. Our strategy is to periodically exchange the staggered fluxes between $\{\phi_1 = \phi, \phi_2 = \pi\}$ and $\{\phi_1 = \pi, \phi_2 = \phi\}$. The condensate wave packet, initially prepared on spinor site A, undergoes unidirectional transfer, ensuring complete transport to the next site A at a specific moment with the appropriate ϕ. Since the π fluxes play a crucial role in constraining

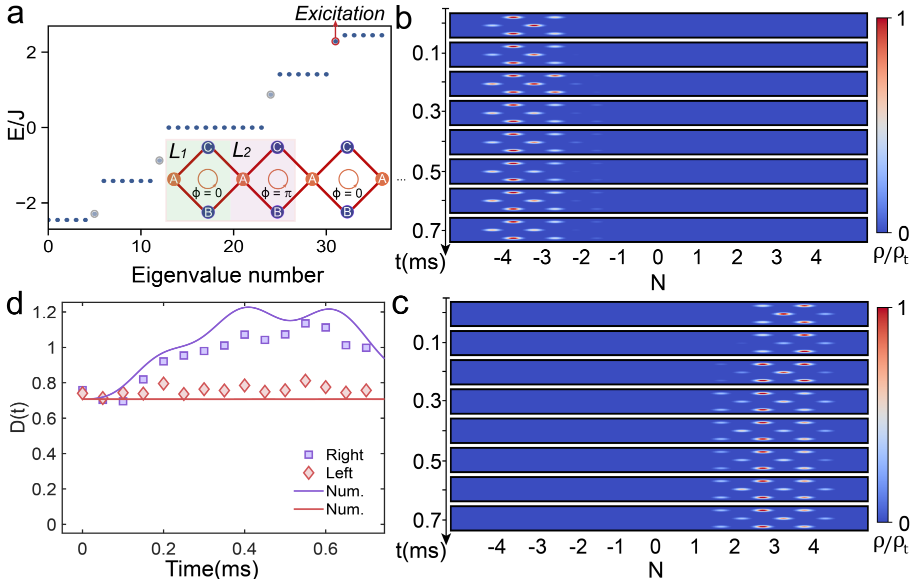

Figure S5: **Detecting the Chiral edge state in the BFL. a.** The quasienergy spectrum of the $\{\phi_1 = 0, \phi_2 = \pi\}$ BFL configuration, where we take a chain with 12 unit cells. There are four isolated eigenvalues that indicate four chiral edge states located at the left edge. **b.** Preparing the top left chiral edge state (highlighted red in (a)) at the left edge side, and observing its evolution dynamics around 0.7 ms. **c.** Symmetrically Preparing edge state with the same form as (b) at the right edge side, and then observing its evolution dynamics. **d.** Comparing the dynamics behaviors in (b) and (c) with $\mathcal{D}(t)$ (here we label the 0_th unit cell at the left/right-most unit cell in (b)/(c). From the data, we can see the well-localized feature of the left chiral edge state.

the condensate's oscillation within one unit cell, the dynamics with fixed staggered fluxes are predominantly governed by the Hamiltonian of symmetric eight sites (in terms of a unit cell with six sites, the four side sites are doubly counted here) surrounding the plaquette with flux ϕ. The eight-site Hamiltonian is given by

$$H_8 = J \begin{bmatrix} 0 & 0 & e^{i\frac{\pi}{4}} & 0 & 0 & 0 & 0 & 0 \\ 0 & 0 & e^{-i\frac{\pi}{4}} & 0 & 0 & 0 & 0 & 0 \\ e^{-i\frac{\pi}{4}} & e^{i\frac{\pi}{4}} & 0 & e^{i\frac{\phi}{4}} & e^{-i\frac{\phi}{4}} & 0 & 0 & 0 \\ 0 & 0 & e^{-i\frac{\phi}{4}} & 0 & 0 & e^{i\frac{\phi}{4}} & 0 & 0 \\ 0 & 0 & e^{i\frac{\phi}{4}} & 0 & 0 & e^{-i\frac{\phi}{4}} & 0 & 0 \\ 0 & 0 & 0 & e^{-i\frac{\phi}{4}} & e^{i\frac{\phi}{4}} & 0 & e^{i\frac{\pi}{4}} & e^{-i\frac{\pi}{4}} \\ 0 & 0 & 0 & 0 & 0 & e^{-i\frac{\pi}{4}} & 0 & 0 \\ 0 & 0 & 0 & 0 & 0 & e^{i\frac{\pi}{4}} & 0 & 0 \end{bmatrix}, \tag{S25}$$

under the basis $\{C_{n-1}, B_{n-1}, A_n, C_n, B_n, A_{n+1}, C_{n+1}, B_{n+1}\}$. Here we have chosen a symmetric gauge

$$\begin{aligned}&\{C_{n-1}, B_{n-1}, A_n, C_n, B_n, A_{n+1}, C_{n+1}, B_{n+1}\} \to \\ &\{\hat{c}_{n-1}, -i\hat{b}_{n-1}, e^{-i\frac{3\pi}{4}}\hat{a}_n, e^{-i(\frac{3\pi}{4}-\frac{\phi}{4})}\hat{c}_n, e^{-i(\frac{3\pi}{4}+\frac{\phi}{4})}\hat{b}_n, e^{-i\frac{3\pi}{4}}\hat{a}_{n+1}, -i\hat{c}_{n+1}, -\hat{b}_{n+1}\},\end{aligned} \tag{S26}$$

to make the forms of Hamiltonian and wave functions more symmetric and simple. Assuming H_8 is diagonalized by the eigenstate matrix $\{|\psi_1\rangle, |\psi_2\rangle, \cdots, |\psi_8\rangle\}$ with eigenvalues $\{\varepsilon_1, \varepsilon_2, \cdots, \varepsilon_8\}$, the time evolution equation $|\psi(t)\rangle = e^{-iH_8 t}|\psi(0)\rangle$ is expanded as $|\psi(t)\rangle = \sum_{l=1}^{8} \alpha_{0l}|\psi_l\rangle e^{-i\varepsilon_l t}$ if $|\psi(0)\rangle = \sum_{l=1}^{8} \alpha_{0l}|\psi_l\rangle$. We find $\varepsilon_3 = \varepsilon_4 = \varepsilon_5 = \varepsilon_6 = 0$ and the third and sixth components of $|\psi_{l=3,4,5,6}\rangle$ are all zeros. It implies $\alpha_{0l=3,4,5,6} = 0$ if the condensate is initially prepared at site A_n.

In contrast, $\varepsilon_1 = -\varepsilon_8 = J\sqrt{2[2+\cos(\phi/2)]}$ and $\varepsilon_2 = -\varepsilon_7 = J\sqrt{2[2-\cos(\phi/2)]}$, and

$$\begin{aligned}|\psi_1\rangle &\propto \{1, -i, (1-i)\sqrt{2+\cos\frac{\phi}{2}}, \sqrt{2}(1-i)\cos\frac{\phi}{4}, \sqrt{2}(1-i)\cos\frac{\phi}{4}, (1-i)\sqrt{2+\cos\frac{\phi}{2}}, -i, 1\}^T, \\ |\psi_8\rangle &\propto \{1, -i, (i-1)\sqrt{2+\cos\frac{\phi}{2}}, \sqrt{2}(1-i)\cos\frac{\phi}{4}, \sqrt{2}(1-i)\cos\frac{\phi}{4}, (i-1)\sqrt{2+\cos\frac{\phi}{2}}, -i, 1\}^T,\end{aligned} \tag{S27}$$

with the same normalization factor $1/[2\sqrt{2(2+\cos\frac{\phi}{2})}]$, and

$$\begin{aligned}|\psi_2\rangle &\propto \{-1, i, (i-1)\sqrt{2-\cos\frac{\phi}{2}}, \sqrt{2}(1+i)\sin\frac{\phi}{4}, -\sqrt{2}(1+i)\sin\frac{\phi}{4}, (1-i)\sqrt{2-\cos\frac{\phi}{2}}, -i, 1\}^T, \\ |\psi_7\rangle &\propto \{-1, i, (1-i)\sqrt{2-\cos\frac{\phi}{2}}, \sqrt{2}(1+i)\sin\frac{\phi}{4}, -\sqrt{2}(1+i)\sin\frac{\phi}{4}, (i-1)\sqrt{2-\cos\frac{\phi}{2}}, -i, 1\}^T,\end{aligned} \tag{S28}$$

with the same normalization factor $1/[2\sqrt{2(2-\cos\frac{\phi}{2})}]$, which dominate the time evolution. Actually, the eigenvalues of real-space Hamiltonian H_8 has the same values as bulk Hmiltonian (S20), i.e., $\varepsilon_1 = E_2$, $\varepsilon_2 = E_1$, $\varepsilon_{3,\ldots,6} = E_0$, $\varepsilon_7 = E_{-1}$, and $\varepsilon_8 = E_{-2}$.

For convenience, by changing the global phases, $|\psi_1\rangle \to |\psi_1\rangle e^{i\pi/4}$, $|\psi_2\rangle \to -|\psi_2\rangle e^{i\pi/4}$, $|\psi_7\rangle \to |\psi_7\rangle e^{i\pi/4}$, $|\psi_8\rangle \to -|\psi_8\rangle e^{i\pi/4}$, we have the simple projections $\langle A_n|\psi_1\rangle = \langle A_n|\psi_2\rangle = \langle A_n|\psi_7\rangle = \langle A_n|\psi_8\rangle = 1/2$ and $\langle A_{n+1}|\psi_1\rangle = -\langle A_{n+1}|\psi_2\rangle = -\langle A_{n+1}|\psi_7\rangle = \langle A_{n+1}|\psi_8\rangle = 1/2$, where $|A_n\rangle = \{0, 0, 1, 0, \cdots, 0\}^T$ and $|A_{n+1}\rangle = \{0, \cdots, 0, 1, 0, 0\}^T$. With these results, the time evolution of condensate wave functions at sites A_n and A_{n+1} when initially preparing the condensate at site A_n, i.e., $|\psi(0)\rangle = |A_n\rangle$, are given by

$$\langle A_n|\psi(t)\rangle = \frac{1}{2}[\cos(\varepsilon_1 t) + \cos(\varepsilon_2 t)], \tag{S29}$$

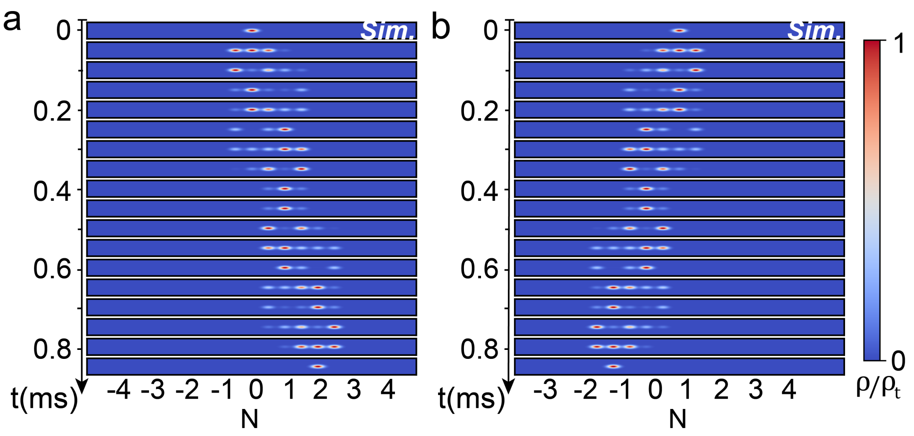

Figure S6: **Simulation of the Floquet channel in the BFL. a.** The numerical result of the Floquet channel with the right chiral transport. **b.** The numerical result of the Floquet channel with the right chiral transport.

and

$$\langle A_{n+1}|\psi(t)\rangle = \frac{1}{2}[\cos(\varepsilon_1 t) - \cos(\varepsilon_2 t)]. \tag{S30}$$

The perfect transfer $|\langle A_{n+1}|\psi(t)\rangle| = 1$ in a certain moment requires

$$\varepsilon_1 T_{\text{opt},\mu\nu}/2 = (\mu+\nu)\pi, \quad \varepsilon_2 T_{\text{opt},\mu\nu}/2 = \mu\pi, \quad \mu = 1,2,3,\cdots, \quad \nu = 1,3,5,\cdots. \tag{S31}$$

The solutions of the above equations are given by

$$\phi_{\text{opt},\mu\nu} = 2\arccos\left[\frac{2\nu(2\mu+\nu)}{\mu^2+(\mu+\nu)^2}\right], \tag{S32}$$

and

$$T_{\text{opt},\mu\nu} = \frac{j\pi}{J\sqrt{2(2-\cos\frac{\phi_{opt,\mu\nu}}{2})}}, \tag{S33}$$

i.e., Eq. (2) and Eq. (3) in the main text,

The preceding discussion establishes the conditions for achieving perfect population transfer within the spinor pair. Under these conditions, the synthetic magnetic flux can induce an effective coupling between the spin-up and spin-down states, arising from the interplay of flat-bands localization and chiral dynamics. The Floquet protocol, as described in the main text, allows us to deduce the Floquet Bloch Hamiltonian $H_k^F = \frac{i}{T}log[U_k(T,0)] = \frac{1}{T}k\sigma_z$. This is achieved by extracting the spectroscopic moments and disregarding the micromotions of other lattice sites during the intermediate moments.

For instance, by exciting different spin components of the spinor pair, we can simulate the Floquet channel dynamics under the effective Hamiltonian (H_{eff}) with the optimal flux pair

($\phi_1 = 0.442\pi, \phi_2 = \pi$). This pair is chosen for its maximal effective coupling strength between the spinor components. The simulation results are depicted in Fig. S6, and the corresponding experimental results are presented in the main text.

VII. Equivalence between winding numbers and transport distances of condensate

In this subsection, we prove the equivalence between the winding numbers of quasi-energy bands and the displacement of condensate, which allows us to detect winding numbers in the experiment by probing the movement of condensate. If the condensate is initially prepared at site 0 of spin σ, under the stroboscopic description, the average position of the condensate is given by

$$x_\sigma(t) = \langle w_{0\sigma}|U^{-1}(t,0)\hat{x}U(t,0)|w_{0\sigma}\rangle, \tag{S34}$$

with the position operator $\hat{x}$ and the initial condensate wave function $|w_{0\sigma}\rangle$ (the Wannier function essentially). The time evolution operator is given by $U(t,0) = \mathcal{T}\exp[-i\hbar^{-1}\int_0^t d\tau H(\tau)]$ with the time-ordering operator $\mathcal{T}$ and the periodic time-dependent Hamiltonian

$$H(t) = \begin{cases} H_1, & 0 \leq \mod(t, T/2) < T/2 \\ H_2, & T/2 \leq \mod(t, T/2) < T. \end{cases} \tag{S35}$$

The eigenstates of the Floquet Hamiltonian $H_k^F = k\sigma_z/T$ are the right- and left-running modes $|\varphi_{k\sigma=\uparrow,\downarrow}\rangle$ with quasienergies $\epsilon_{k\sigma}$. We thus have

$$|w_{0\sigma}\rangle = \frac{1}{\sqrt{2\pi}}\int_{-\pi}^{\pi} dk|\varphi_{k\sigma}\rangle, \tag{S36}$$

since the Floquet Hamiltonian is decoupling in the quasimomentum space and has no off-diagonal terms [see Eq. (4) of the main text]. Inserting the completeness relation of $|\varphi_{k\sigma}\rangle$ into Eq. (S34), we have

$$\begin{aligned} x_\sigma(t) =& \sum_{\gamma\gamma'}\int_{-\pi}^{\pi}\int_{-\pi}^{\pi} dkdk' \langle w_{0\sigma}|U^{-1}(t,0)|\varphi_{k\gamma}\rangle\langle\varphi_{k\gamma}|\hat{x}|\varphi_{k'\gamma'}\rangle\langle\varphi_{k'\gamma'}(0)|U(t,0)|w_{0\sigma}\rangle \\ =& d\sum_{\gamma}\int_{-\pi}^{\pi} dk\langle w_{0\sigma}|U^{-1}(t,0)|\varphi_{k\gamma}\rangle i\partial_k\langle\varphi_{k\gamma}|U(t,0)|w_{0\sigma}\rangle \\ =& d\sum_{\gamma}\int_{-\pi}^{\pi} dk\langle w_{0\sigma}|u_{k\gamma}(t)\rangle e^{-i\epsilon_{k\sigma}t} i\partial_k e^{i\epsilon_{k\sigma}t}\langle u_{k\gamma}(t)|w_{0\sigma}\rangle, \end{aligned} \tag{S37}$$

where $|u_{k\gamma}(t)\rangle$ is the periodic function satisfying $|u_{k\gamma}(t)\rangle = |u_{k\gamma}(t+T)\rangle$ (9), and d is the lattice constant of the staggered-flux lattice, i.e., $d = 2$ according to the units in the main text.

Considering $|u_{k\gamma}(T)\rangle = |u_{k\gamma}(0)\rangle = |\varphi_{k\sigma}\rangle$, we have

$$x_\sigma(T) = \frac{d}{2\pi}\sum_\gamma \int_{-\pi}^{\pi} dk e^{-i\epsilon_{k\sigma}T} i\partial_k e^{i\epsilon_{k\sigma}T}. \tag{S38}$$

Thus the transport distance after one driving period, $x_\sigma(T)$, is nothing but $\nu_\sigma d$, by noting that the quasienergy $\epsilon_{k\sigma=\uparrow\downarrow} = \pm\Omega k/2\pi = \pm k/T$. This equivalence relation between winding number and transport distance has been reported previously (see Ref. [45] in the main text). It means that the transport distance of the condensate is proportional to the winding number of the corresponding quasi-energy band on which the condensate is initially prepared. Following the same route, we have

$$\begin{aligned}\mathcal{D}_\sigma(T) &= \langle w_{0\sigma}|U^{-1}(T,0)\hat{x}^2 U(T,0)|w_{0\sigma}\rangle^{1/2} \\ &= (-d^2\frac{1}{2\pi}\int_{-\pi}^{\pi} dk e^{-i\epsilon_{k\sigma}T}\partial_k^2 e^{i\epsilon_{k\sigma}T})^{1/2}.\end{aligned} \tag{S39}$$

It follows that $|\mathcal{D}(T)|$ ($\mathcal{D}(T) = \pm\mathcal{D}_{\sigma=\uparrow,\downarrow}(T) = x_{\sigma=\uparrow,\downarrow}(T)$ for the rightward and leftward motion) in Fig. 3**h** gives the experimental value of $|\nu_\sigma|d$.

References

1. B. Gadway, *Phys. Rev. A* **92**, 043606 (2015)

2. E. J. Meier, F. A. An and B. Gadway, *Phys. Rev. A* **93**, 051602 (2016)

3. H. Li, Z. Dong, S. Longhi, Q. Liang, D. Xie, and B. Yan, *Phys. Rev. Lett.* **129**, 220403 (2022)

4. Supplementary/ Material: Aharonov-Bohm Caging and Inverse Anderson transition in Ultracold Atoms [2022]; American Physical Society; https://doi.org/10.1103/PhysRevLett.129.220403

5. Harry Levine, Alexander Keesling, et.al., *Phys. Rev. Lett.* **121**, 123603 (2018)

6. Eric J Meier, Fangzhao Alex An, et.al., *Science* **362**, 929-933 (2018)

7. Yunfei Wang, Jia-Hui Zhang, et.al., *Phys. Rev. Lett.* **129**, 103401 (2022)

8. Chao Zeng, Yue-Ran Shi, et.al., *Phys. Rev. Lett.* **132**, 063401 (2024)

9. Martin Holthaus, *Journ. of Phys. B: Atom., Mol. and Opt. Phys.* **49**, 013001 (2015)
